# Editorial Correspondence

**Published:** 1877-09

**Authors:** I. H. Goss

**Affiliations:** Fort Lamar, Ga.


FORT LAMAR, GA., Sept. 13, 1877.

*Messrs. Editors Atlanta Medical and Surgical Journal:*

Having met with a case recently which I deem an interesting one, I decide to give you a short history of the same, and if you consider it of sufficient importance, you may give it a place in your journal.

The case is a young man, aged twenty-one years, who is suffering from epilepsy, caused by a wound he received while laboring in a field, on January 17, 1869, by the falling of a tree, which struck him on the head and fractured the frontal bone, a knot entering the brain. A physician was sent for, who came, examined the case, but failing to discover the piece of wood, dressed the wound and left. One hundred and thirty-three days from the time of the accident a hard substance was discovered by the father of the young man, near the surface. Drs. Mathews, of Elbert county, Ga., were sent for, and extracted a knot 1½ inches long. Twenty-one days thereafter a piece of a woolen hat and lining was taken from the wound. The depressed bone was soon removed, the wound healing readily. In October, 1871, nearly three years after the injury, and over two years after removal of the foreign substance from the fracture, he was attacked with epilepsy, which has continued to this day.

I saw him August 24, 1877, and remained with him about thirty minutes only, during which time he had six spasms or convulsions. Could an operation for the removal of the depressed bone be of any benefit to him at this late date, eight years after receiving the injury? I would be very glad to hear from any one on this subject.

Yours very truly, I. H. Goss, M.D.

We think the foregoing case is one in which trephining is certainly demanded. The operation has been performed with great benefit when the point of injury to the cranium could not be satisfactorily ascertained, and indeed when the only explanation of relief that could be given was that of counter-irritation or allowing a little more space for the brain. In this case, doubtless a spicula of bone, a piece of wood or other substance in contact with the meninges or brain-substance becomes a source of constant irritation, leading to the convulsions. Notwithstanding the unnatural adhesions that may be expected, and the difficulty and danger of removing the irritating cause, the case certainly requires an attempt at relief by removal of a portion of bone, including the depressed part. In the patient's present condition, he will certainly drag through a miserable and useless existence, and an operation will most assuredly leave him with no darker prospects for future usefulness and pleasure, than surround him at present. Moreover, it would seem unreasonable to expect relief from this most distressing disease from any other means than removal of the local irritating cause, at the same time using brain tonics to break up the habit.—[Editor.

---

